# Contact-free assessments of respiratory rate and volume with load cells under the bed legs in ventilated patients: a prospective exploratory observational study

**DOI:** 10.1152/japplphysiol.00742.2022

**Published:** 2023-04-20

**Authors:** Azusa Inada, Shin Inaba, Yosuke Matsumura, Takuya Sugiyama, Noriyuki Hanaoka, Naohiko Fujiyoshi, Natsuko Nozaki-Taguchi, Yasunori Sato, Shiroh Isono

**Affiliations:** ^1^Department of Anesthesiology, Graduate School of Medicine, Chiba University, Chiba, Japan; ^2^Department of Anesthesiology, Chiba Emergency Medical Center, Chiba, Japan; ^3^Department of Intensive Care, Chiba Emergency Medical Center, Chiba, Japan; ^4^Department of Preventive Medicine and Public Health, Keio University School of Medicine, Tokyo, Japan

**Keywords:** centroid, load cell, respiration, respiratory rate, tidal volume

## Abstract

Development of reliable noncontact unrestrained respiratory monitoring is capable of augmenting the safety of hospitalized patients in the recovery phase. We previously discovered respiratory-related centroid shifts along the long axis of the bed with load cells under the bed legs [bed sensor system (BSS)]. This prospective exploratory observational study examined whether noncontact measurements of respiratory-related tidal centroid shift amplitude (TA-BSS; primary variable) and respiratory rate (RR-BSS; secondary variable) were correlated with tidal volume (TV-PN) and respiratory rate (RR-PN), respectively, measured by pneumotachograph in 14 ICU patients under mechanical ventilation. Among the 10-min average data automatically obtained for a 48-h period, 14 data samples were randomly selected from each patient. Successfully and evenly selected 196 data points for each variable were used for the purpose of this study. A good agreement between TA-BSS and TV-PN (Pearson’s *r* = 0.669) and an excellent agreement between RR-BSS and RR-PN (*r* = 0.982) were observed. Estimated minute ventilatory volume [3.86 · TA-BSS · RR-BSS (MV-BSS)] was found to be in very good agreement with true minute volume (MV-PN) (*r* = 0.836). Although Bland–Altman analysis evidenced accuracy of MV-BSS by a small insignificant fixed bias (−0.02 L/min), a significant proportional bias of MV-BSS (*r* = −0.664) appeared to produce larger precision (1.9 L/min) of MV-BSS. We conclude that contact-free unconstrained respiratory monitoring with load cells under the bed legs may serve as a new clinical monitoring system, when improved.

**NEW & NOTEWORTHY** We previously discovered that four load cells placed under the bed legs capture a centroid shift during respiration in bedridden human subjects. In 14 ICU patients under mechanical ventilation, this study evidenced that contact-free measurements of respiratory rate, tidal volume, and minute ventilation with the load cells correlated well with those measured by pneumotachograph. Possible clinical usefulness of this approach as a new clinical respiratory monitor is indicated.

## Graphical abstract

**Figure:**
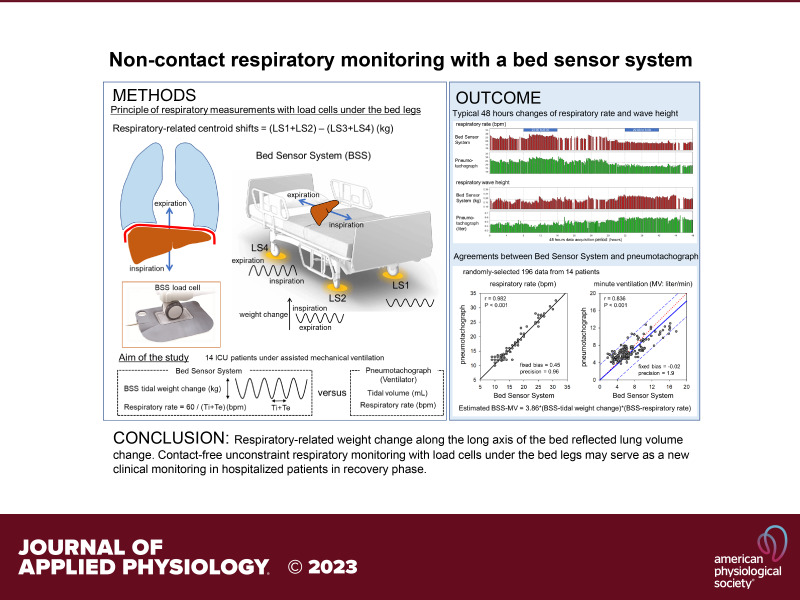

## INTRODUCTION

Respiration is a fundamental vital sign reflecting health and disease conditions and is generally characterized by its rhythmicity and magnitude. Accurate assessments of respiration are of great significance, particularly in hospitalized patients at risk of deterioration of respiratory as well as nonrespiratory disorders (1, 2). Although a patient monitoring system is used for treatment and management in the acute phase, the wires and cables of the equipment disturb patients’ mobilization and are disconnected in the recovery phase to facilitate early mobilization and rehabilitation for prevention of disuse muscle atrophy and sarcopenia (3). However, acute deterioration during the recovery period is not rare, particularly during the nursing handover time and nighttime, when patient observation resources are reduced (2, 4, 5). Increase of the respiratory rate is reported to be the most important predictor and indicator for deterioration (1). Furthermore, frequency of periodic tidal volume oscillation describes the nature and severity of Cheyne–Stokes respiration and obstructive sleep apnea and hypopnea well (6). Irregularity of both respiratory rhythmicity and magnitude is a characteristic feature of ataxic breathing, often identified in patients receiving high-dose opioid treatment (7). In this context, the development of reliable noncontact unrestrained respiratory monitoring is capable of augmenting the safety of hospitalized patients with minimum nurse workload increase.

In our recent study, we reported a system with respiratory-related centroid shifts along the long axis of the bed with load cells under the bed legs. Through use of the unconstrained Bed Sensor Vital-Sign Monitoring System (BSS), the accuracy of respiratory rate measurements was validated in healthy awake adult volunteers (8). Potential values of its application to health and disease managements have been suggested (9), and in fact we succeeded in identifying bradypnea and respiratory rhythm irregularity in advanced cancer patients receiving opioid without contact or restriction of their daily activities (7). Under careful observation of BSS amplitude changes, as well as respiratory rhythm, accurate diagnosis of abnormal respiratory patterns, such as Cheyne–Stokes respiration, obstructive sleep disordered breathing, and ataxic breathing, is possible. However, despite the potential usefulness of the BSS respiratory waves as respiratory pattern monitoring for patients recovering from a critical condition, no systematic assessment of association between BSS respiratory wave amplitude and tidal volume has been performed to date. Accordingly, as a validation step before assessing the clinical role of BSS, this study aimed to test a hypothesis that BSS tidal amplitude (TA-BSS; primary variable) and BSS respiratory rate (RR-BSS; secondary variable) reflect tidal volume (TV-PN) and respiratory rate (RR-PN) measured by a ventilator pneumotachograph in ICU patients under mechanical ventilation. Similarly, a comparison was made between RR-PN and respiratory rate measured by the clinically most commonly used impedance pneumography from a patient monitoring system (RR-IP) as a reference to RR-BSS.

## METHODS

### Ethics and Setting

This prospective observational study was performed at Chiba Emergency Medical Center, Chiba, Japan. Ethical approval for this study (Ethical Committee number: 1440) was provided by the Ethical Committee of Chiba Emergency Medical Center on March 11, 2022. The study was registered before patient enrollment at the UMIN (University Hospital Information Network) Clinical Registry: (UMIN000047793; principal investigator: Azusa Inada, date of registration: May 18, 2022, website: https://center6.umin.ac.jp/cgi-open-bin/ctr_e/ctr_view.cgi?recptno=R000054491). Contact information for the full trial information is available on the UMIN website. Because of the safe noncontact feature of the BSS, measurements were started immediately after ICU admission. The investigators fully explained the aim and potential risks of the study to each patient or the patient’s relatives at the appropriate time. Only data of patients for whom written informed consent was obtained from the patients or their relatives were used in this study.

### Participants

Subject enrollments in this study were started on July 20, 2022 and terminated on November 21, 2022. Inclusion criteria were adult patients who were mechanically ventilated because of acute respiratory failures or other medical reasons at the intensive care unit of Chiba Emergency Medical Center, where critically ill emergency patients are transported by ambulance. Original exclusion criteria in this study were patients treated with artificial organs other than the ventilator and those treated on a bed on which installment of load cells was not possible. However, during data acquisition of this study, although a number of the enrolled patients were revealed to have had treatments with artificial organs, respiratory waves of these patients were able to be confirmed. Accordingly, to minimize the number of participants and use all available data for the purpose of this study, the ethical committee certified inclusion of patients treated with artificial organs. In accordance with the study protocol, we approached 29 patients and relatives, and written informed consent was obtained from 20 patients. Six patients were excluded from the analysis because of cessation of mechanical ventilation within 24 h.

### Patient Care and Treatments

Patient care and treatments including cardiorespiratory management were routinely performed based on the ICU protocol and discussions of their clinical conditions at daily ICU staff meetings, without any bias. Vital signs were continuously monitored by a patient monitoring system (BSM-6701 Life ScopeTR, BSM-1763 Life ScopePT; Nihon Kohden, Tokyo, Japan) integrating information of electrocardiogram, thoracic impedance pneumography, invasive arterial pressure, body temperature, capnogram, and pulse oximetry. All patients were intubated with either 7.5 (female)- or 8.0 (male)-mm-inner diameter (ID) tracheal tube for mechanical ventilation therapy with an advanced adult ventilator (Puritan Bennet 980 and 840, Medtronic, Dublin, Ireland; VELA, Vyaire Medical, Mettawa, IL). The ventilator setting was individually determined based on arterial blood gas analysis and vital signs. Each 1-min vital sign data sample from the patient monitoring system and respiratory data from the ventilator were stored on the patient data monitoring server (PRM-7100 Prime GAIA; Nihon Kohden).

Patients stayed on the bed for the study period, excluding the transfer time during patient transfer for diagnostic imaging and invasive procedures outside the ICU. Appropriate types of ICU electrical beds (Multicare ALT, LINET, Slaný, Czech Republic; Enterprise 9000X, Arjo, Malmö, Sweden; Versacare, Hill-Rom, Chicago, IL; KA-75120A, Paramount bed, Tokyo, Japan) allowing (automatic) body position changes and body weight measurement with a pressure-relief air mattress (OSCAR MOSC91, Molton, Hiroshima, Japan) were selected for each patient depending on severity and necessity. In general, the patients were placed in the supine position with a 30° bed head angulation, except for patients in the shock state. Sedatives were continuously infused and adjusted during the acute phase of mechanical ventilation therapy throughout the day. The patients were given bed baths at least once a day, and an early mobilization protocol was actively applied, although these activities on the bed by nurses and physical therapists may have contributed to interference with BSS monitoring.

### Measurements of Respiratory Variables

Respiratory variables measured by the ICU ventilator [respiratory rate (RR-PN), tidal volume (TV-PN)] and impedance pneumography from the patient monitoring system [respiratory rate (RR-IP)] were extracted from the data servers. Accuracy of tidal volume measurements by the ventilators was reported to be ±1.0–10 mL; ±10%. Forty-eight hours of per-minute data starting at 2 PM on the first day under mechanical ventilation were averaged at 10-min intervals and used for the analyses. RR-PN and TV-PN measured by pneumotachograph inside the ventilator were considered to be gold standard values to be compared with the BSS respiratory variables.

### Respiratory Variables Measured by Noncontact BSS with Load Cells under the Bed Legs

The BSS vital sign monitoring system (a BSS prototype; MinebeaMitsumi Inc., Nagano, Japan) consists of four high-resolution strain gauge load cell sensors [C2G1-50K-A: rated output (RO) 2.0 ± 0.2 mV/V, rated capacity 50 kg, total error 0.02% RO; MinebeaMitsumi Inc.] placed under the legs of the ICU bed and a data logger for processing and analyzing the load cell signals. The under-bed BSS continuously captures total weight on the sensors and movements on the bed including body movements and respiratory-related centroid shifts (7, 8). Each load cell sensor independently measured weight, and the digital load cell signal was sampled at 200 Hz and filtered for each load cell before decimation (10 Hz) for data analyses.

As reported previously, load cell signals of the upper (LC3, LC4) and lower (LC1, LC2) sensors correlated to respiratory-related shifts of the abdominal visceral organs along the long axis of the bed (*y*-axis) (8). Accordingly, the composite wave *Y* = (LC1 + LC2) − (LC3 + LC4) presenting changes of the centroid along the *y*-axis was considered to reflect respiration and was used for contact-free respiratory assessments. BSS uses the composite wave to calculate both the breath-by-breath and 25-s moving average of RR-BSS and TA-BSS after excluding body movement portions of the wave. All signals and variables were processed at 10-Hz sampling rate within the data logger and wirelessly sent to a local server personal computer comprised of automatically generating CSV files, including mean or median values of the variables for each 10-min interval, through use of data analyzing software. The measurements were processed automatically without involvement of either nurses or investigators, and the data were later downloaded from the local server for the analyses.

### Outcomes of the Study and Sample Size Calculation

The primary outcome is to test the hypothesis that TA-BSS (primary variable) and RR-BSS (secondary variable) reflect TV-PN and RR-PN, respectively, during mechanical ventilation. The hypothesis was evaluated by the value of Pearson’s correlation coefficient (*r*) between the variables, and *r* > 0.7 was considered to be physiologically and clinically meaningful (10). To expect *r* > 0.7 under assumptions of α = 0.05, β = 0.8, the required sample size was 14 participants.

### Statistical Analysis

Variables are expressed as frequencies and proportions for categorical data and as means and standard deviations (SDs) for continuous variables. Although up to 288 samples of the 10-min average data were maximally obtained for the 48 h, an unspecified large number of missing data were expected, particularly for BSS variables because of sustained body movements on the bed. To minimize effects of the patient factor on the results of the statistical analysis, 14 data samples were randomly selected from each patient by the simple random sampling without replacement method and used for the following statistical analyses. Correlation between variables was assessed by Pearson’s correlation coefficient and a linear regression analysis. Bland–Altman analysis was performed to assess agreements of RR-BSS and RR-IP to RR-PN. To estimate minute ventilatory volume corresponding MV-V = RR-PN × TV-PN (L·min^−1^) with RR-BSS and TA-BSS, the ratio of mean RR-BSS × TA-BSS to mean RR-PN × TV-PN was used. Statistical analyses were performed with statistical software (SigmaPlot 13.0; Systat Software Inc., Point Richmond, CA). *P* values were two-sided, and a value of *P* < 0.05 was considered statistically significant.

## RESULTS

Data acquisition was successfully completed in 14 Japanese patients (12 males, 2 females) mechanically ventilated with various mechanical ventilation modes (Tables 1 and 2). Participants demonstrated heterogeneous age, body features, and critical illness characteristics. Notably, most patients were managed with heavy high-performance beds (*n* = 13) with air mattresses (*n* = 10). No harmful events associated with placement of the load cells under the bed legs and the study protocol occurred. Eight patients had invasive surgeries or catheter interventions before ICU admission. Eight patients were treated with artificial organs during the BSS measurements. Although muscle relaxant was not used, sedatives were used in 12 patients. Ventilator settings allowed spontaneous breathing under ventilator-assisted respiration.

**Table 1.** Patients' background variables

Anthropometrics	
Sex (male, female)	2, 12
Age, yr	66.3 ± 15.5
Height, m	1.64 ± 0.11
Body weight, kg	71.0 ± 19.8
Body mass index, kg/m^2^	26.1 ± 5.8
Indications for ICU managements	
Cardiac diseases	7 (50)
Septic shock	2 (14)
Trauma	2 (14)
Postresuscitation	1 (7)
Hepatorenal syndrome	1 (7)
Subarachnoidal hemorrhage	1 (7)
Invasive treatments before ICU admission	
Cardiac surgeries	4 (29)
Cardiac catheter interventions	2 (14)
Coil embolization	1 (7)
Forearm reconstruction surgery	1 (7)
Artificial organs used during the study	
Intra-aortic balloon pumping	5 (36)
Continuous renal replacement therapy	3 (20)

Values are frequency (proportion) or mean ± SD.

**Table 2.** Cardiorespiratory conditions and managements

Ventilator modes	
Assist/control-pressure control	9 (64)
Assist/control-volume control	4 (29)
Spontaneous	1 (7)
Sedatives and muscle relaxant	
Midazolam	7 (50)
Propofol	5 (36)
Dexmedetomidine	3 (20)
Fentanyl	12 (86)
Rocuronium	0 (0)
None	2 (14)
Ventilator settings and blood gases at beginning of measurements	
Fraction of inspired oxygen	0.47 ± 0.20
PEEP, cmH_2_O	7.0 ± 2.1
Arterial blood gas: pH	7.42 ± 0.06
Arterial blood gas: $\text{P}{\text{a}}_{{\text{O}}_{2}}$, mmHg	138 ± 96
Arterial blood gas: $\text{P}{\text{a}}_{\text{C}{\text{O}}_{2}}$, mmHg	36.9 ± 7.0
Ratio of $Pa_{O_{2}}$ to fraction of inspired oxygen	295 ± 104
Vital signs at beginning of measurements	
Systolic blood pressure, mmHg	100 ± 17
Diastolic blood pressure, mmHg	55 ± 6
Heart rate, beats/min	99 ± 23
$\text{S}{\text{a}}_{{\text{O}}_{2}}$, %	98 ± 1
Glasgow Coma Scale	4.6 ± 2.4

Values are frequency (proportion) or mean ± SD. $\text{P}{\text{a}}_{{\text{O}}_{2}}$, arterial partial pressure of oxygen; $\text{P}{\text{a}}_{\text{C}{\text{O}}_{2}}$, arterial partial pressure of carbon dioxide; PEEP, positive end-expiratory pressure; $\text{S}{\text{a}}_{{\text{O}}_{2}}$, arterial oxygen saturation.

**Figure 1.**
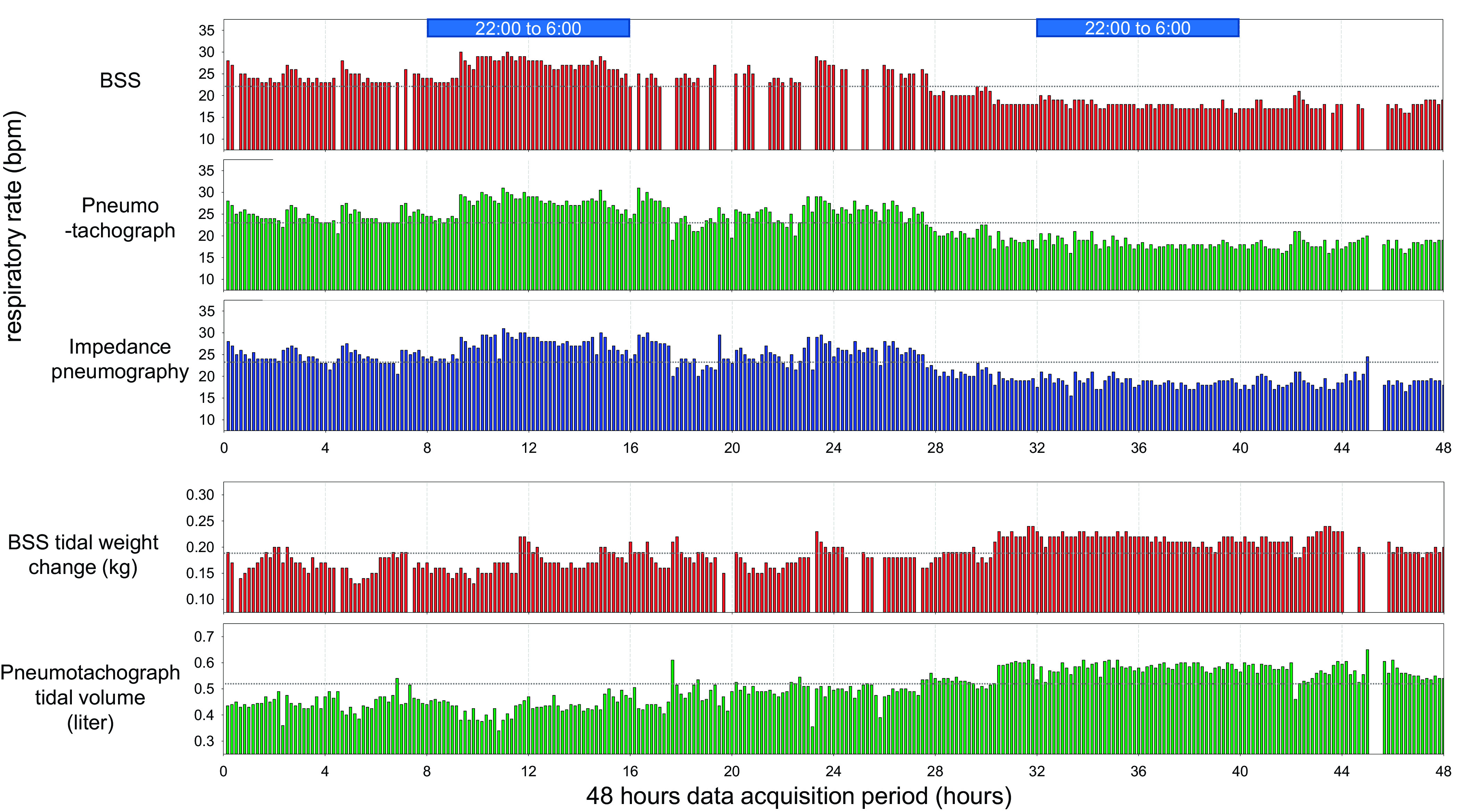
Changes of respiratory variables for a 48-h data acquisition period in a 73-year-old male patient with septic shock (body wt 81 kg). Respiratory variables were measured by the unconstrained Bed Sensor Vital-Sign Monitoring System (BSS), the pneumotachograph of the ventilator, and impedance pneumography of the patient monitoring system. Each bar denotes a 10-min average value of the respiratory variable. Note agreements among 3 different techniques for measurement of respiratory rate (*top*) and agreements of trends between BSS tidal weight change and tidal volume measured by pneumotachograph (*bottom*). bpm, Breaths/min.

Figure 1 presents changes of respiratory variables for the 48-h data acquisition period in a 73-yr-old male patient with septic shock (body wt 81 kg). Neither sedatives nor muscle relaxants were used, and spontaneous breathing mode was applied. The patient was managed on a high-performance bed (219 × 100 cm, 250 kg) with an air mattress (13 kg). Changes of the respiratory rate measured by BSS are in almost complete agreement with those measured by the ventilator pneumotachograph. Stabilization of the respiratory rate on the second day, after a relatively unstable respiratory rate at the start of data acquisition, was well captured by all three different techniques of respiratory rate measurement. The pattern of respiratory tidal weight changes was also in good agreement with changes of tidal volume measured by the pneumotachograph. In accordance with the decrease of respiratory rate on the second day, increase of the tidal volume for maintenance of minute ventilation was also well described by the BSS tidal weight change.

### Characteristics of the Respiratory Variables and Performance of Data Acquisition

Table 3 presents respiratory variables automatically obtained in the 48-h data acquisition period. Compared with the respiratory variables measured by the pneumotachograph, BSS had significantly greater percentages of missing data, >20% of the data acquisition period, because of body movements and interventions by medical staff. The randomly selected respiratory variables (*n* = 196) well reflected those obtained from all participants.

**Table 3.** Respiratory variables obtained for 48 h

	All Data (max. 4,032 data points)				Randomly Selected Data (*n* = 196)	
	Data points	% Missing	Mean ± SD	95% CI	Mean ± SD	95% CI
Pneumotachograph (PN) in ventilator					
RR-PN, bpm	3,745	7.1	15.5 ± 5.1	15.4–15.7	15.2 ± 5.0	14.5–16.0
TV-PN, L	3,688	8.5	0.46 ± 0.09	0.45–0.46	0.46 ± 0.10	0.44–0.47
MV-PN, L/min	3,688	8.5	6.98 ± 2.36	6.90–7.06	6.80 ± 2.10	6.51–7.10
BSS						
RR-BSS, bpm	3,129	22.4*	14.4 ± 4.6	14.3–14.6	14.8 ± 4.9	14.1–15.5
TA-BSS, kg	3,414	15.3*	0.13 ± 0.05	0.12–0.13	0.13 ± 0.05	0.12–0.14
RR-BSS × TA-BSS, kg/min	3,122	22.6	1.80 ± 0.90	1.76–1.83	1.86 ± 0.89	1.73–2.00
Estimated MV-BSS, L/min	3,122	22.6*	6.59 ± 3.31	6.48–6.71	6.82 ± 3.3	6.36–7.29
Impedance pneumography (IP)						
RR-IP, bpm	3,857	4.3*	16.2 ± 5.3	16.0–16.4	15.7 ± 5.1	15.0–16.4

bpm, Breaths/min; BSS, bed sensor system ; CI, confidence interval; MV, minute ventilatory volume; PN, RR, respiratory rate; TA, tidal centroid shift amplitude; TV, tidal volume. *P < 0.05 vs. the variables measured by the ventilator.

### Primary Outcome of the Study: Association of Respiratory Variables Measured by BSS and Pneumotachograph

**Figure 2.**
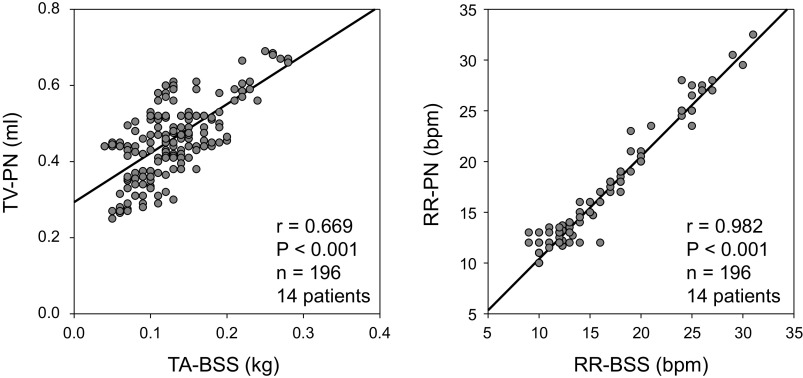
Associations between tidal weight change measured by the contact-free Bed Sensor Vital-Sign Monitoring System (TA-BSS) and tidal volume measured by pneumotachograph (TV-PN) (*left*) and between respiratory rate measured by BSS (RR-BSS) and respiratory rate measured by pneumotachograph (RR-PN) (*right*). Fourteen data points were randomly selected from the 10-min average data obtained for a 48-h period in each patient, and 196 data points from 14 patients were used to determine the Spearman correlation coefficient (*r*). bpm, Breaths/min.

Successfully and evenly selected 196 data points for each variable from 14 patients (Table 3) were used for testing the primary hypothesis that TA-BSS (primary variable) and RR-BSS (secondary variable) measured by BSS reflect TV-PN and RR-PN measured by the ventilator pneumotachograph during mechanical ventilation. As illustrated in Fig. 2, statistically significant direct associations between TA-BSS and TV-PN and between RR-BSS and RR-PN were evident. Pearson’s *r* value between TA-BSS and TV-PN (*r* = 0.669) was <0.7, although indicating good agreement enough for qualitative and semiquantitative assessment of lung volume change during breathing by BSS. Pearson’s *r* value between RR-BSS and RR-PN (*r* = 0.986) indicated excellent agreement enough for quantitative assessment of respiratory rate by BSS.

### Agreements of Respiratory Variables Measured by BSS and Impedance Pneumography with Those Measured by Pneumotachograph in the Ventilator

**Figure 3.**
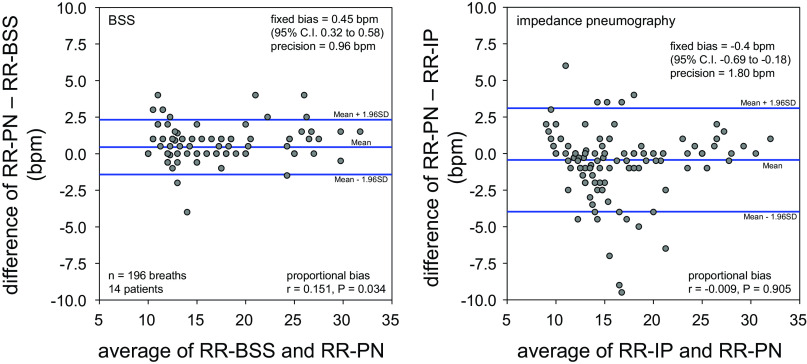
Results of Bland–Altman analyses of respiratory rate measured by the contact-free Bed Sensor Vital-Sign Monitoring System (RR-BSS; *left*) and respiratory rate measured by impedance pneumography (RR-IP; *right*) in comparison with respiratory rate measured by pneumotachograph (RR-PN). bpm, Breaths/min; CI, confidence interval.

Figure 3 presents results of Bland–Altman analyses of respiratory rate measured by BSS and impedance pneumography. For a relatively wide range of respiratory rates, both BSS and impedance pneumography had clinically acceptable accuracy and precision, whereas the precision of RR-BSS [0.96 breaths/min (bpm)] was half of that of RR-IP (1.80 bpm). RR-BSS and RR-IP had small but significant positive and negative fixed bias, respectively.

### Prediction Model for Minute Ventilatory Volume by BSS

**Figure 4.**
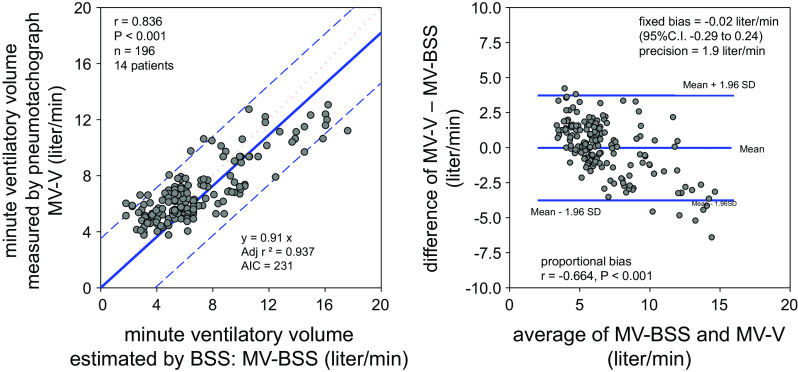
Results of Brand–Altman analysis of minute ventilatory volume estimated by the contact-free Bed Sensor Vital-Sign Monitoring System (MV-BSS) and minute ventilatory volume measured by pneumotachograph (MV-PN). AIC, Akaike information criterion; CI, confidence interval.

With TA-BSS and RR-BSS, minute ventilatory volume (MV-BSS) was estimated, and its accuracy and precision were assessed by Bland–Altman analysis (Fig. 4). A very good agreement between MV-BSS and MV-V was observed, as indicated by the high Pearson’s *r* value (*r* = 0.836). The prediction model had excellent features of a linear regression model. Furthermore, Bland–Altman analysis evidenced accuracy of MV-BSS by the small insignificant fixed bias. However, a relatively larger precision value indicated a large deviation of MV-BSS from MV-V. In fact, BSS accurately estimated only 49% and 40% of 196 data points within ±20% and ± 1 L/min of MV-V, respectively. At best, 72% and 73% of MV-BSS data were within ±30% and ± 2 L/min of MV-V, respectively. The large deviation occurred at higher and lower ranges of minute ventilatory volume, possibly because of a significant proportional bias of MV-BSS (*r* = −0.664).

## DISCUSSION

In this prospective exploratory observational study performed in ICU patients under mechanical ventilation, a good agreement was observed between TA-BSS (primary variable) measured by load cells under the bed legs and TV-PN measured by ventilator pneumotachograph (*r* = 0.669), whereas an excellent agreement between RR-BSS (secondary variable) and RR-PN (*r* = 0.982) was observed. Performance of MV-BSS for estimation of MV-V was promising, but its nature of a proportional bias produced substantial deviations at lower and higher ranges of minute ventilatory volume.

### Contributions of Respiratory-Related Shifts of the Center of Gravity along the Long Axis of the Bed to Lung Volume Change

Results of this study clearly supported our hypothesis of association between respiratory-related weight change along the log axis of the bed and lung volume change. This hypothesis is based on the assumption of pistonlike movement in an elliptic cylinder representing lung volume increase during quiet breathing (11). Mathematically, the volume change of the elliptic cylinder (V) is a function of the short-axis radius (*a*), the long-axis radius (*b*), and the height (*h*) (V = π*abh*). When the elliptic thoracic cage shape is constant, the lung volume linearly increases with increase in the cranio-caudal diaphragm movement distance during breathing. Mead and Loring (12) demonstrated greater diaphragm contribution to tidal volume than rib cage contribution. Recently, Wang et al. (13) demonstrated a linear association between diaphragm shift and lung volume change, predominantly at the abdominal compartment, during spontaneous breathing. In our study, direct association between respiratory-related weight shifts along the long axis of the bed and lung volume change also agreed with the pistonlike movement of the diaphragm and the abdominal visceral organs along the long axis during breathing. However, the associations between them were not parallel, and a significant proportional bias of the lung volume estimation by respiratory-related weight shifts existed, causing deviation from the true lung volume change (Fig. 4). Although the mechanisms and causes of the proportional bias are unclear at present, both physiological as well as technical aspects of the possibilities need to be explored, and an improved prediction model with BSS variables needs to be developed in the near future for clinical application.

### Clinical Implications of the Study Results: Potential for a Contact-Free Respiratory Monitor

Development of a contact-free respiratory monitor is challenging but would serve for significant improvement of hospitalized patient safety and public health promotion, as Schaefer and Eikermann (9) suggested. There are growing interests and efforts in development of contact-free respiratory monitors, particularly after COVID-19; however, most of these studies tested only respiratory rates and did not assess tidal volumes. Furthermore, they were performed in nonclinical situations and for very limited short periods, under supervision of the researchers, when the best performance of the devices was possible (14–18). Depth camera and radar have been reported to produce excellent performance in measuring tidal volume without subject contact of human volunteers; however, clinical implementation of these technologies appears to be difficult because of difficulty in maintaining different body positions of patients covered by blankets (14–17). To our knowledge, only one study has systematically assessed performance of tidal volume measurements with load cells under the bed legs (18). Jung et al. (18) used a globalized machine learning-based algorithm, including both respiratory and cardiac signals, for estimating tidal volume in 15 healthy adult volunteers undergoing respiratory tasks in four different body positions. They reported a Pearson’s *r* = 0.85 and root mean square error (RMSE) = 0.23 L, results that were comparable to our study although our study found a large deviation at lower and higher tidal volume estimations and obvious proportional bias. In our study, the respiratory variables were automatically measured for 48 h in ICU patients under sedation, after which they were sent to the server and analyzed without researchers’ attendance. Although there was 20% missing data of BSS monitoring, we consider it acceptable since nurses only routinely check respiration every 2–3 h in patients in whom the regular patient monitoring system is no longer necessary. Although this is only the initial step in understanding the role of BSS in the assessment of recovering patients and much more work is necessary, load cells under the bed legs appear to have promising features and performance of respiratory assessments as a reliable clinical respiratory monitor with minimum increase of nurse workload in the near future.

### Limitations of the Study and Issues to Be Addressed

There are several methodological limitations of this study. First, patients were mechanically ventilated and the respiratory centroid shifts measured by the load cells may be different from those in patients spontaneously breathing since diaphragm movements during spontaneous breathing differ significantly from those during mechanical ventilation under anesthesia and paralysis (19). This may be responsible for the proportional bias of MV estimation by BSS. Second, time spent in care and treatment of the patients, which may have influenced the respiratory variable measurements, was not recorded and considered for the analysis. However, we considered it reasonable to assess the performance under the routine of clinical management. Third, 10-min average data were used for the analysis, whereas clinicians expect more timely reporting of patient vital signs, which the conventional monitor equipment provides. Since body movements have a tendency of interfering with BSS respiratory monitoring, subsequently resulting in substantial missing data as was detected even in patients under sedation, BSS respiratory monitoring is considered to be less useful for patients in the acute phase, since vital sign deterioration is highly anticipated and requires immediate response by medical staff. In contrast, for patients in the recovery phase, nurses routinely check the vital signs every 2–3 h in many hospitals, although only 20% of hospitals include respiratory rate in routine vital sign check monitoring (20). Early warning signs, such as increase of respiratory rate, are reported to precede cardiac arrest by 5–6 h (21, 22). Accordingly, reporting accurate vital signs every 10 min may significantly reduce unmonitored time and improve hospitalized patient safety.

In conclusion, respiratory-related weight change along the long axis of the bed reflected lung volume change, and contact-free unconstrained respiratory monitoring with load cells under the bed legs may serve as a new mode of clinical monitoring in hospitalized patients in recovery phase.

## DATA AVAILABILITY

Data will be made available upon reasonable request.

## GRANTS

This study was supported by the Joint Research Expenses from MinebeaMitsumi Inc.

## DISCLOSURES

This study used data obtained during collaborative research between Chiba University and MinebeaMitsumi Inc.. S. Isono received scholarship donations from MinebeaMitsumi Inc. as a head of Department of Anesthesiology, Chiba University. S. Isono is one of the inventors of the vital sign monitoring system used in this study. None of the other authors has any conflicts of interest, financial or otherwise, to disclose.

## AUTHOR CONTRIBUTIONS

A.I., S. Inaba, N.N.-T., Y.S., and S. Isono conceived and designed research; A.I., S. Inaba, Y.M., T.S., N.H., and N.F. performed experiments; A.I., Y.S., and S. Isono analyzed data; A.I., S. Inaba, N.N.-T., Y.S., and S. Isono interpreted results of experiments; A.I. prepared figures; A.I., Y.S., and S. Isono drafted manuscript; N.N.-T., Y.S., and S. Isono edited and revised manuscript; A.I., S. Inaba, Y.M., T.S., N.H., N.F., N.N.-T., Y.S., and S. Isono approved final version of manuscript.

## References

[1] Parr MJ, Hadfield JH, Flabouris A, Bishop G, Hillman K. The medical emergency team: 12 month analysis of reasons for activation, immediate outcome and not-for-resuscitation orders. Resuscitation 50: 39–44, 2001. doi:10.1016/S0300-9572(01)00323-9. 11719127

[2] Dumot JA, Burval DJ, Sprung J, Waters JH, Mraovic B, Karafa MT, Mascha EJ, Bourke DL. Outcome of adult cardiopulmonary resuscitations at a tertiary referral center including results of “limited” resuscitations. Arch Intern Med 161: 1751–1758, 2001. doi:10.1001/archinte.161.14.1751. 11485508

[3] Gustafsson UO, Scott MJ, Hubner M, Nygren J, Demartines N, Francis N, Rockall TA, Young-Fadok TM, Hill AG, Soop M, de Boer HD, Urman RD, Chang GJ, Fichera A, Kessler H, Grass F, Whang EE, Fawcett WJ, Carli F, Lobo DN, Rollins KE, Balfour A, Baldini G, Riedel B, Ljungqvist O. Guidelines for Perioperative Care in Elective Colorectal Surgery: Enhanced Recovery After Surgery (ERAS®) Society Recommendations: 2018. World J Surg 43: 659–695, 2019. doi:10.1007/s00268-018-4844-y. 30426190

[4] Jones D, Bellomo R, Bates S, Warrillow S, Goldsmith D, Hart G, Opdam H, Gutteridge G. Long term effect of a medical emergency team on cardiac arrests in a teaching hospital. Crit Care 9: R808–R815, 2005. doi:10.1186/cc3906. 16356230 PMC1414057

[5] Jones D, Bates S, Warrillow S, Opdam H, Goldsmith D, Gutteridge G, Bellomo R. Circadian pattern of activation of the medical emergency team in a teaching hospital. Crit Care 9: R303–R306, 2005. doi:10.1186/cc3537. 16137341 PMC1269438

[6] Redline S, Budhiraja R, Kapur V, Marcus CL, Mateika JH, Mehra R, Parthasarthy S, Somers VK, Strohl KP, Sulit LG, Gozal D, Wise MS, Quan SF. The scoring of respiratory events in sleep: reliability and validity. J Clin Sleep Med 3: 169–200, 2007. 17557426

[7] Hasegawa M, Nozaki-Taguchi N, Shono K, Mizuno Y, Takai H, Sato Y, Isono S. Effects of opioids on respiration assessed by a contact-free unconstraint respiratory monitor with load cells under the bed in patients with advanced cancer. J Appl Physiol (1985) 130: 1743–1753, 2021. doi:10.1152/japplphysiol.00904.2020. 33886386

[8] Isono S, Nozaki-Taguchi N, Hasegawa M, Kato S, Todoroki S, Masuda S, Iida N, Nishimura T, Noto M, Sato Y. Contact-free unconstraint respiratory measurements with load cells under the bed in awake healthy volunteers: breath-by-breath comparison with pneumotachography. J Appl Physiol (1985) 126: 1432–1441, 2019. doi:10.1152/japplphysiol.00730.2018. 30763161

[9] Schaefer MS, Eikermann M. Contact-free respiratory monitoring using bed wheel sensors: a valid respiratory monitoring technique with significant potential impact on public health. J Appl Physiol (1985) 126: 1430–1431, 2019. doi:10.1152/japplphysiol.00198.2019. 30920886

[10] Byrt T. How good is that agreement? Epidemiology 7: 561, 1996. doi:10.1097/00001648-199609000-00030. 8862998

[11] DeTroyer A, Loring SH. Action of the respiratory muscle. In: Handbook of Physiology: The Respiratory System, edited by Macklem PT, Mead J. Bethesda, MD: American Physiological Society, 1986, p. 443–461.

[12] Mead J, Loring SH. Analysis of volume displacement and length changes of the diaphragm during breathing. J Appl Physiol Respir Environ Exerc Physiol 53: 750–755, 1982. doi:10.1152/jappl.1982.53.3.750. 6215387

[13] Wang HK, Lu TW, Liing RJ, Shih TT, Chen SC, Lin KH. Relationship between chest wall motion and diaphragmatic excursion in healthy adults in supine position. J Formos Med Assoc 108: 577–586, 2009. doi:10.1016/S0929-6646(09)60376-4. 19586832

[14] Oh K, Shin CS, Kim J, Yoo SK. level-set segmentation-based respiratory volume estimation using a depth camera. IEEE J Biomed Health Inform 23: 1674–1682, 2019. doi:10.1109/JBHI.2018.2870859. 30235149 PMC7309325

[15] Addison PS, Smit P, Jacquel D, Addison AP, Miller C, Kimm G. Continuous non-contact respiratory rate and tidal volume monitoring using a depth sensing camera. J Clin Monit Comput 36: 657–665, 2022. doi:10.1007/s10877-021-00691-3. 33743106 PMC7980749

[16] Islam SM. Radar-based remote physiological sensing: progress, challenges, and opportunities. Front Physiol 13: 955208, 2022. doi:10.3389/fphys.2022.955208. 36304581 PMC9592800

[17] Massagram W, Lubecke VM, Boric-Lubecke O. Microwave non-invasive sensing of respiratory tidal volume. Annu Int Conf IEEE Eng Med Biol Soc 2009: 4832–4835, 2009. doi:10.1109/IEMBS.2009.5332667. 19963859

[18] Jung H, Kimball JP, Receveur T, Gazi AH, Agdeppa ED, Inan OT. Estimation of tidal volume using load cells on a hospital bed. IEEE J Biomed Health Inform 26: 3330–3341, 2022. doi:10.1109/JBHI.2022.3141209. 34995200

[19] Froese AB, Bryan AC. Effects of anesthesia and paralysis on diaphragmatic mechanics in man. Anesthesiology 41: 242–255, 1974. doi:10.1097/00000542-197409000-00006. 4604401

[20] Cardona-Morrell M, Prgomet M, Lake R, Nicholson M, Harrison R, Long J, Westbrook J, Braithwaite J, Hillman K. Vital signs monitoring and nurse-patient interaction: A qualitative observational study of hospital practice. Int J Nurs Stud 56: 9–16, 2016. doi:10.1016/j.ijnurstu.2015.12.007. 26775214

[21] Schein RM, Hazday N, Pena M, Ruben BH, Sprung CL. Clinical antecedents to in-hospital cardiopulmonary arrest. Chest 98: 1388–1392, 1990. doi:10.1378/chest.98.6.1388. 2245680

[22] Franklin C, Mathew J. Developing strategies to prevent in hospital cardiac arrest: analyzing responses of physicians and nurses in the hours before the event. Crit Care Med 22: 244–247, 1994. 8306682

